# Investigation of ossification in the posterior longitudinal ligament using micro-focus X-ray CT scanning and histological examination

**DOI:** 10.1186/s13000-015-0440-8

**Published:** 2015-11-21

**Authors:** Katsunori Fukutake, Takao Ishiwatari, Hiroshi Takahashi, Kazuaki Tsuchiya, Yoichiro Okubo, Minoru Shinozaki, Naobumi Tochigi, Megumi Wakayama, Tetsuo Nemoto, Kazutoshi Shibuya, Akihito Wada

**Affiliations:** Department of Orthopedic Surgery, Toho University Omori Medical Center, 6-11-1, Omori-Nishi, Ota-Ku, Tokyo, 143-8541 Japan; Department of Surgical Pathology, Toho University School of Medicine, 6-11-1, Omori-Nishi, Ota-Ku, Tokyo, 143-8541 Japan; Department of Dermatology, Peking University First Hospital, Beijing, China

**Keywords:** Ossification of the posterior longitudinal ligament, Micro-focus X-ray CT, Mechanisms of ossification

## Abstract

**Background:**

Ossification in the posterior longitudinal ligament (PLL) correlates with changes of enthesis during the early stages of development, but this issue remains controversial, as little is known regarding the details of this process. The aim of the present study was to elucidate part of the ossification mechanism. Thus, in the present study, we observed and evaluated minute ossifications in the PLL that did not exhibit symptoms of ossification of the posterior longitudinal ligament (OPLL).

**Methods:**

The subjects in the present study were derived from serial autopsy cases from January 2009 to December 2013 at Toho University Omori Medical Center, Japan. Minute ossifications in the PLL from autopsy subjects without any history of OPLL were screened as high-density areas using micro-focus X-ray CT, and the foci were histologically examined. Subsequently, we conducted both micro-focus X-ray CT image analysis and histological examination, and evaluated the correlation between these findings and putative predictive factors reported in previous studies.

**Results:**

A total of 103 individuals among the 267 subjects involved in the present study were analyzed within the study period. There were no cases involving OPLL identification prior to death, and no subjects presented with neurological symptoms of myelopathy. The incidence of cases involving high-density areas greater than 0.1 mm^2^ in the PLL was 46.6 %, half of which revealed mature bone structures inside this area. Thus, the high-density areas comprised three types: a continuous posterior-annular fibrosus type (23 cases), an isolated posterior-annular fibrosus type (11 cases), and a posterior-vertebral type (29 cases). However, a positive correlation was observed between the proportion of high-density areas, age (Pearson *r* = 0.265, *p* < 0.01), and HbA1c (Pearson *r* = 0.294, *p* < 0.01). Histological examination confirmed that these high-density areas involved calcification with or without mature bone formation.

**Conclusions:**

We evaluated minute foci of calcification with and without ossification in the PLL from 103 cadavers, generating the following observations:

1. Minute calcification foci greater than 0.1 mm^2^ were observed in the PLL of 48 cases (46.6 %), half of which revealed mature bone structures inside this area (23.3 %).
2. The proportion of minute calcification foci observed in the present study was correlated with age and glucose tolerance, suggesting changes in the OPLL in the early stage.
3. Three different mechanisms of ossification were suggested: The two structures developed behind the disc might reflect the elongation of enthesis or rupture of annular fibrosus, while the remaining structure developed behind the vertebral body might reflect a dystrophic calcification-based bony metaplasia sequence.

## Background

Ossification of the posterior longitudinal ligament (OPLL) is a refractory disease initially reported [1] in 1938, which causes compressive myelopathy. Many subsequent studies were conducted to investigate the process of ossification, and there is increasing evidence of ossification factors involving age, gender [2], body mass index (BMI) [3], insulin [4], glucose intolerance [5], myotonic dystrophy [6], and endocrine diseases (vitamin D-resistant rickets [7], parathyroid function [8]). Histological examinations of OPLL have revealed hyperplasia in fibrous cartilage tissue continuous with the annulus fibrosus of the intervertebral disc, denaturation of the ligament in the area near the enthesis and the metamorphosis to cartilaginous tissue, with calcification and metaplasia to mature bone tissue within these areas of cartilaginous tissue [9]. A close relationship between the ossification of the posterior longitudinal ligament and enthesis during the early stages of enthesopathy has been proposed [10], but this issue remains controversial.

The purpose of the present study was to elucidate part of the ossification mechanism. We observed minute ossifications in the posterior longitudinal ligament (PLL) of autopsy subjects that did not exhibit symptoms of OPLL. For this purpose, we employed a micro-focus X-ray CT system (inspeXio SMX-100CT, Shimadzu Corporation Tokyo, Japan) to generate extensive sequential tomography images and performed the histological examination of the characteristic sections. Furthermore, we evaluated the correlation between the present findings and putative predictive factors of ossification previously reported in autopsy cases.

## Methods

### Subject

The subjects were derived from serial autopsy cases recorded between January 2009 and December 2013 at Toho University Omori Medical Center, Japan. Cases in which the spinal columns had not been detected and the PLL was not suitable for observation were excluded. The protocol for the present study was approved through the Ethics Committee of the Toho University School of Medicine (#260242600825093).

### Specimen preparation

**Fig. 1:**
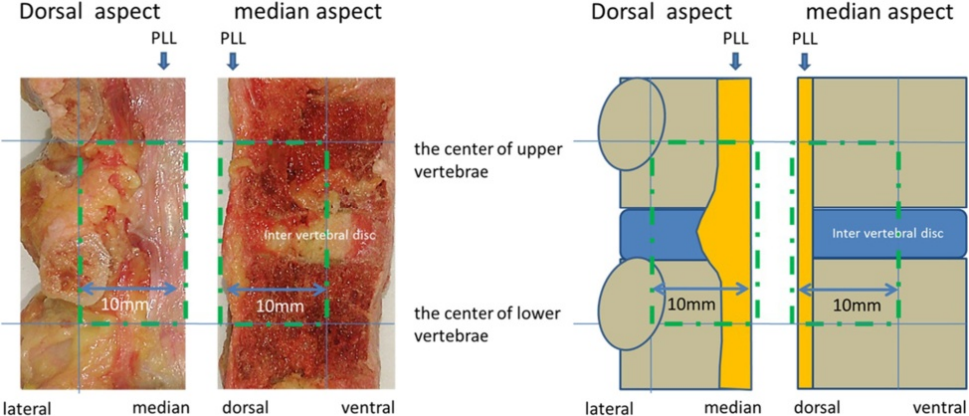
Methods of trimming the specimens. These figures show the trimmed line. The specimens were trimmed 1 cm ventrally from the dorsal vertebra and 1 cm laterally from the midline of the cross-section

The spinal columns from the autopsy specimens were divided in half at the midline sagittal plane from Th10 to L5, including eight vertebrae and seven intervertebral levels and fixed with formalin. A previous study reported that the L1/L2 level contains the highest frequency of OPLL occurring in thoracolumbar spinal columns [11]; therefore, two vertebrae above and below this level, including the L1/L2 intervertebral disc, were amputated at the center of each vertebral body and excised. To examine a midline sagittal cross-section view of the PLL, the specimens were trimmed 1 cm ventrally from the dorsal vertebra and 1 cm laterally from the midline of the cross-section (Fig. 1).

### Image analysis using non-distraction micro-focus X-ray CT scanning

**Fig. 2:**
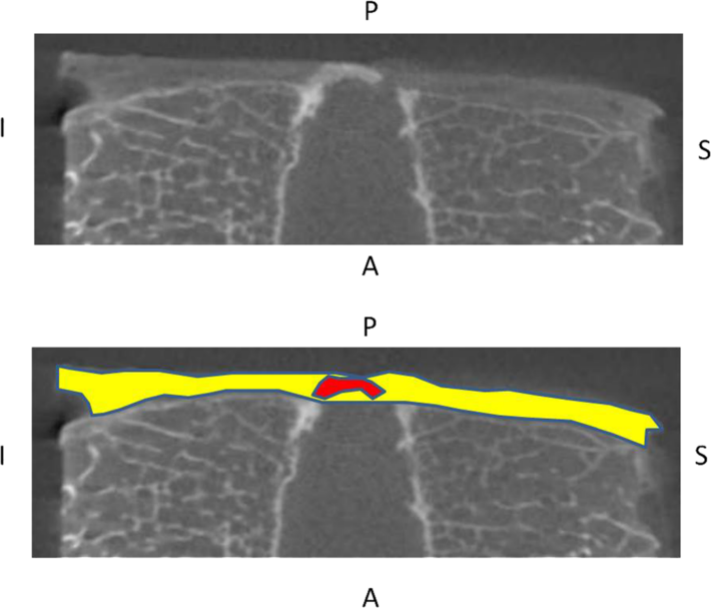
Measuring methods used to analyze the two-dimensional tomogram images. This figure shows the measuring methods utilized to analyze the two-dimensional tomogram images using image analysis software, showing a slice of the two-dimensional tomogram images as an example. Red area: high-density area with a density equal to or greater than that of vertebral bone trabeculae in an area equal to or greater than 0.1 mm^2^. Yellow area: the soft tissue behind vertebrae as the PLL. The boundary between the PLL and the annular fibrosus of the intervertebral disc were defined as the line connecting the top of the rim of the upper and lower vertebrae

The present study employed a micro-focus X-ray CT scanning system (inspeXio SMX-100CT Shimadzu Corporation, Tokyo, Japan), with the maximum tube voltage and maximum tube current adjusted to 75 kV and 160 μA, respectively. Sequential tomography images of each sample were obtained with 16-bit 512 × 512 pixel resolution. After 30 serial slices of 0.1 mm in width were obtained from the sagittal plane, the slices were reconstructed into a sagittal section image. Two-dimensional tomogram images were analyzed using image analysis software (Image processing and analysis in JAVA 1.48v; National Institutes of Health, Bethesda, State of Maryland, USA). Additionally, an area of ossification and calcification in the PLL was evaluated as a high-density area as demonstrated through micro-focus X-ray CT, defined as a density equal to or greater than that of vertebral bone trabeculae in an area equal to or greater than 0.1 mm^2^ (Fig. 2). The evaluation points included the frequency of detection of the high-density area, form of the high-density area, mean and maximum posterior longitudinal ligament thickness, sharpness of the rim, and the proportion of the high-density area (the percentage of the total area of each slice of high-density areas divided by the total area of each slice of the PLL). Three-dimensional reconstruction was also performed using ray casting based on three-dimensional volume rendering software (VG Studio Max 2.2, Volume Graphics, Germany) with 30 serial slices of each specimen. The evaluation points were confirmed from the high-density areas, and the continuity with bones was observed.

### Extraction of clinical information concerning putative predictive factors of ossification

To evaluate the correlation between the findings obtained through micro-focus CT image analysis and previously reported putative predictive factors for ossification, details of the medical history of each case, including the cause of death, age at death, gender, body-mass index and hemoglobin A1c (HbA1c) value at the time of final admission, past history of myotonic dystrophy, vitamin D-resistant rickets, hypoparathyroidism, and diabetes, which might affect ossification, were obtained after evaluating the medical and autopsy records.

### Histological examinations

Following decalcification, each specimen was transected and embedded in paraffin. A thin section was obtained from the embedded block, which represented the midline sagittal plane and included the high-density area observed through micro-focus X-ray CT, and subsequently stained with hematoxylin and eosin. We observed forms of ossification and calcification in detail and assessed changes in the tissue near these foci using an optical microscope. The rate of ossification in the total area of calcification was calculated to determine the extent of bone metaplasia among the calcified area.

### Statistical analysis

The statistical analysis was performed using SPSS Statistics software (version 22, IBM, NY, USA). Age, BMI, and HbA1c were analyzed using the *t*-test, and the gender, and prevalence of diabetes were analyzed using the chi-square test. The potential correlation between the putative predictive factors of ossification and the high-density area ratio was analyzed using the Pearson correlation coefficient. The putative predictive factors of ossification for each separate ossification pattern were analyzed using the Mann–Whitney *U*-test.

## Results

### Subjects and clinical background

A total of 267 autopsy cases were recorded at Toho University Omori Medical Center, Japan, within a retrospective observation period. Among these, 164 cases were excluded due to predetermined conditions, and a final group comprising 103 cases was analyzed. This group included 69 males and 34 females aged 32–94 years, with a mean age of 68.5 years. The age distribution is shown in Table 1 (Table 1). There were no cases involving OPLL identification prior to death, and none of the subjects presented neurological symptoms related to myelopathy. The cause of death was solid cancer in 40 cases, infection in 18 cases, hematological malignancy in 15 cases, interstitial pneumonia in eight cases, heart disorder in six cases, and other factors in 19 cases. There were only two cases with hypoparathyroidism, and no subjects exhibited myotonic dystrophy or vitamin D-resistant rickets.

**Table 1:** Age distribution of the subjects

Age	Male	Female	Total
30–39	1	1	2
40–49	5	3	8
50–59	7	4	11
60–69	22	9	31
70–79	21	10	31
80–89	13	6	19
90–99	0	1	1

### Micro-focus X-ray CT image analysis

#### Prevalence

**Fig. 3:**
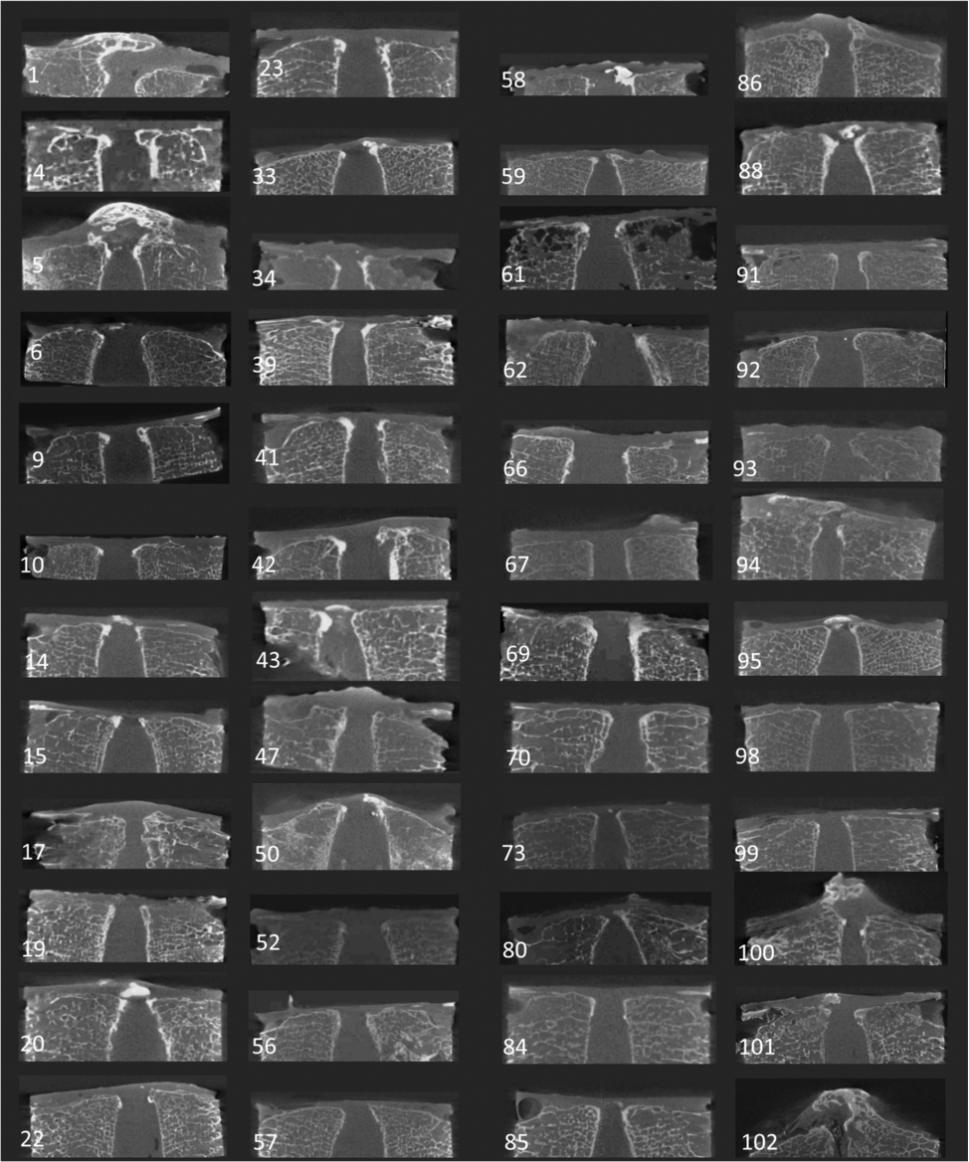
A total of 48 cases with high-density areas in the PLL. The number represents the serial number of the present study

Among the 103 cases analyzed, a high-density area was observed in the PLL of 48 cases (46.6 %) (Fig. 3), comprising 31 males and 17 females with a mean age of 72.4 years, ranging from 41 to 89 years.

#### Structure of ossification (high-density areas)

The structure and form of the high-density areas observed through micro-focus X-ray CT was divided into two categories: the posterior region of annular fibrosus and the posterior region of vertebra. Herein, we describe these locations of high-density areas as posterior-annular fibrosus and posterior-vertebral types, respectively. Although the former category was further subdivided into two subtypes, namely, with or without continuity with vertebra, no cases of the latter category that exhibited continuity with existing bone were observed. Herein, we describe these types as continuous or isolated, respectively.

**Fig. 4:**
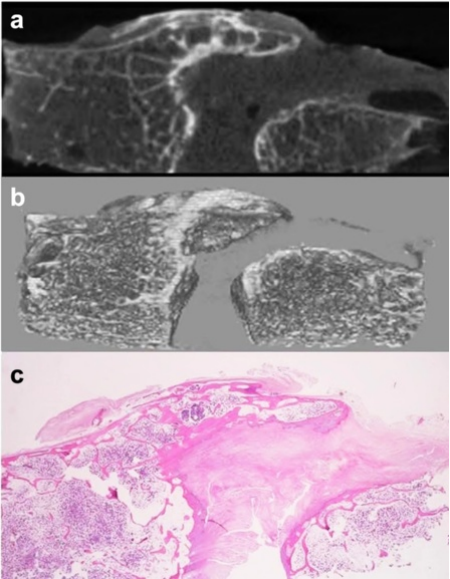
**a**–**c** Representative case of the continuous posterior-annular fibrosus type. **a** CT image: A trabecular bone structure was frequently observed within the high-density area. **b** Three dimensional reconstruction image: The high-density area was observed as an elongation of the rim of the vertebral body. **c** Pathological image: These cases typically involved a projection with continuity with both marrow and the cortex of the bone to the enthesis. Calcification was observed in the connective tissue around the projection

In the continuous posterior-annular fibrosus type, the high-density area was observed as an elongation of the rim of the vertebral body, as a trabecular bone structure was frequently observed within the high-density area and the proportion of the high-density area was high (Fig. 4a and b).

**Fig. 5:**
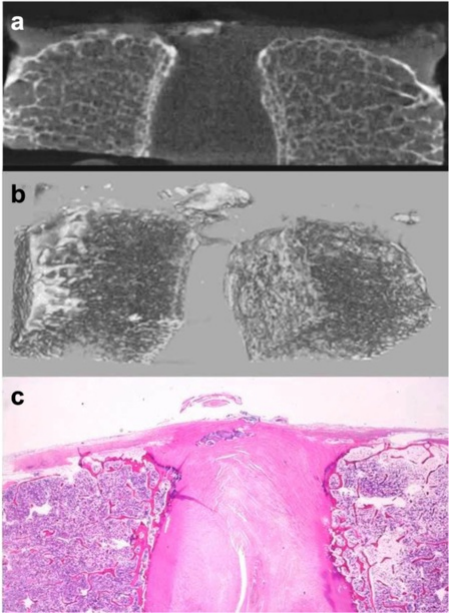
**a**–**c** Representative case of the isolated posterior-annular fibrosus type. **a** CT image: The high-density area was interpreted as a collection of small lesions instead of a single high-density area. **b** Three dimensional reconstruction image: The high-density area was present at isolated intervertebral levels and had no continuity to the vertebra. **c** Pathological image: These cases typically showed the prolapse of degenerated nucleus pulposus with previous rupturing of annular fibrosus of the intervertebral disc, accompanied by fibrosis, calcification and/or ossification

In the isolated posterior-annular fibrosus type, the high-density area was present at isolated intervertebral levels and could be interpreted as a collection of small lesions instead of a single high-density area (Fig. 5a and b).

**Fig. 6:**
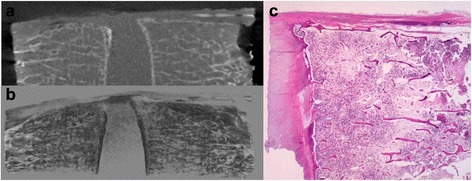
**a**–**c** Representative case of the posterior-vertebral type. **a** CT image: The ligament remained nearly unchanged with regard to thickness and surface area. Localized high-density areas were observed along the longitudinal axis in the PLL. **b** Three dimensional reconstruction image: High-density areas were without continuity with the vertebra. **c** Pathological image: This type showed isolated calcifying foci characterized by a linear-shaped, stereoscopically plate-shaped calcification and/or ossification in the PLL developed just behind the vertebral body

In the posterior-vertebral type, the ligament remained nearly unchanged with regard to thickness and surface area. Localized high-density areas were observed along the longitudinal axis in the PLL, and the proportion of high-density area was relatively low (Fig. 6a and b).

The continuous posterior-annular fibrosus type was observed in 23 cases, the isolated posterior-annular fibrosus type was observed in 11 cases, and the posterior-vertebral type was observed in 29 cases. Among these cases, overlapping forms included the continuous posterior-annular fibrosus type and posterior-vertebral type in 11 cases and the isolated posterior-annular fibrosus type and posterior-vertebral type in four cases (Table 2 and Fig. 3).

**Table 2:** Micro-focus X-ray CT image analysis and histological examination

Case			Micro-focus X-ray CT image analysis				Histological findings
No.	Age	Sex	Proportion of HDA (%)	Type of HDA			Rate of ossification in total area of calcification (%)
				C-PAF	I-PAF	PV	
1	89	F	32.39	+	-	-	98.8
4	81	M	2.31	+	-	+	98.7
5	74	F	50.84	+	-	-	99.8
6	85	M	9.31	-	+	+	0
9	82	M	5.26	+	-	+	0
10	70	F	0.18	+	-	-	0
14	71	M	6.43	-	+	+	0
15	74	M	6.04	+	-	+	0
17	77	M	1.78	-	-	+	0
19	89	F	2.72	-	-	+	65.2
20	65	M	5.35	-	+	-	0
22	69	F	0.08	-	+	-	0
23	68	F	0.36	+	-	+	97.4
33	67	F	1.11	+	-	-	0
34	78	M	5.4	-	+	+	0
39	74	M	7.19	+	-	+	72.9
41	75	M	8.22	+	-	+	39.3
42	61	F	10.91	+	-	+	98.1
43	78	F	10.88	-	+	-	0
47	79	M	2.1	-	-	+	50.8
50	80	F	11.73	+	-	-	0
52	74	M	2.26	-	-	+	0
56	81	M	2.52	-	-	+	0
57	70	M	0.11	+	-	+	78.9
58	59	F	8.27	+	-	-	0
59	64	M	1.89	+	-	-	99.1
61	83	M	3.3	-	-	+	6.3
62	41	M	0.29	+	-	-	0
66	61	M	1.69	-	-	+	0
67	62	M	0.54	-	-	+	0
69	84	M	8.57	+	-	+	81.7
70	78	M	1.13	-	-	+	0
73	81	M	0.64	-	+	+	0
80	82	M	3.03	+	-	-	87.9
84	61	M	2.79	-	-	+	33.7
85	68	M	0.09	-	+	-	0
86	57	M	4.04	+	-	-	99.6
88	52	F	4.82	-	+	-	4.1
91	61	M	1.87	-	-	+	0
92	76	M	9.58	-	+	-	89.9
93	74	M	1.38	-	-	+	0
94	77	F	25.45	+	-	+	99.5
95	49	M	4.51	-	+	-	27.4
98	58	M	1.44	-	-	+	26
99	83	M	3.19	-	-	+	0
100	77	F	0.15	+	-	-	44
101	89	M	4.28	+	-	+	53.9
102	86	M	38.51	+	-	-	96.6

*HDA* high-density area

*C*-*PAF* continuous posterior-annular fibrosus type

*I*-*PAF* isolated posterior-annular fibrosus type

*PV* posterior-vertebra type

### Relationship with putative predictive ossification factors

A baseline comparative investigation was performed using cases having a ossification observed as a high density area in CT image (ossification + group) and those without a ossification (ossification- group) (Table 3). Age and HbA1c were significantly greater in the ossification + group, but there were no significant differences for other factors, such as gender, BMI, past history of diabetes, and presence of malignant tumor. Furthermore, a positive correlation was observed between the proportion of the ossification and age (Pearson *r* = 0.265, *p* < 0.01) and HbA1c (Pearson *r* = 0.294, *p* < 0.01) (Table 4). However, neither gender nor diabetes was associated with statistical significance. The median values of ages with a continuous posterior-annular fibrosus type and posterior vertebral type were higher than those in subjects without the two types (Table 5).

**Table 3:** Comparison between the group with no ossification and the group showing ossification (Mann–Whitney *U*-test)

	All cases	Ossification		r
		(+)	(-)	
Number of cases	103	48	55	-
Age (years)	68.5	72.4	65.1	*p* < 0.01
Sex (male %)	67.0	70.8	63.6	n.s.
BMI (Kg/m2)	21.4	22.1	20.8	n.s.
HbA1c (%)	5.8	6.1	5.5	*p* < 0.01
Diabetes (%)	28.2	31.3	25.5	n.s.
Malignant tumor (%)	58.3	56.3	60.0	n.s.

*BMI* body mass index

**Table 4:** Correlation between the proportion of ossification and/or calcification and putative predictive factors (Pearson correlation coefficient)

		Proportion of O/C	Age	BMI	HbA1c
Proportion of HDA	r	-	0.265	0.062	0.294
	p	-	0.007	0.548	0.004
Age	r	0.265	-	0.105	0.053
	p	0.007	-	0.311	0.607
BMI	r	0.062	0.105	-	0.058
	p	0.548	0.311	-	0.590
HbA1c	r	0.294	0.053	0.058	-
	p	0.004	0.607	0.590	-

*O*/*C* ossification and/of calcification

**Table 5:** Examination of the morphological characteristics of the ossification, the posterior-annular fibrosus type, and the posterior-vertebral type (*t*-test)

	C-PAF	Other	*P* value	I-PAF	Other	*P* value	PV	Other	*P* value
	*n* = 23	*n* = 80		*n* = 11	*n* = 92		*n* = 29	*n* = 74	
Age (years)	73.09	67.20	0.041	70.18	68.32	0.622	74.86	66.03	0.000
BMI (Kg/m^2^)	23.74	20.75	0.034	19.84	21.58	0.115	21.18	21.46	0.767
HbA1c (%)	5.93	5.76	0.315	6.209	5.746	0.151	5.967	5.734	0.344

*C*-*PAF* continuous posterior-annular fibrosus type

*I*-*PAF* isolated posterior-annular fibrosus type

*PV* posterior-vertebra type

### Histological findings

Histological examination through micro-focus X-ray CT analysis revealed comprised calcification foci in high-density areas all cases, and half of the cases showed mature bone structures inside this area (Table 2 and Fig. 3). Moreover, the ratio of bone in the high-density area increased with increasing proportion of the high-density area (Pearson *r* = 0.481 *p* = 0.001).

**Fig. 7:**
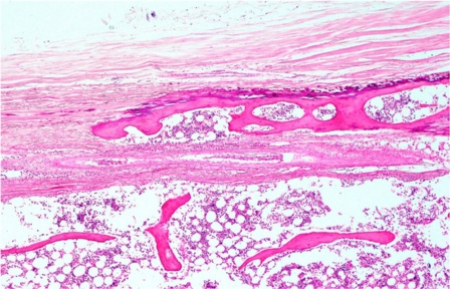
Mature bone tissue in isolated calcifying foci in the PLL. The posterior-vertebral type showing isolated calcifying foci in the PLL, which contained islets of mature bone tissue

In the continuous posterior-annular fibrosus type, the cases likely involved a projection exhibiting continuity with the both marrow and the cortex of the bone to the enthesis, and calcification was observed in the connective tissue around this projection (Fig. 4c). However, the isolated posterior-annular fibrosus type typically showed a prolapse of degenerated nucleus pulposus with previous rupturing of annular fibrosus of the intervertebral disc, accompanied by fibrosis, calcification, and/or ossification (Fig. 5c). The posterior-vertebral type showed isolated calcifying foci in the PLL, some of which contained islets of mature bone tissue (Figs. 6c and 7).

## Discussion

Although OPLL is one of the most contractile diseases in Japan, the detailed pathogenesis of this disease remains controversial. To further elucidate the mechanisms underlying this disease, a detailed investigation of the minute foci of ossification in the PLL of individuals without clinical manifestation of OPLL is needed. Therefore, in the present study, we investigated the prevalence and form of minute foci from autopsies of individuals without OPLL prior to death. In addition, no previous studies have reported the use of micro-focus X-ray CT and 3D reconstruction image analysis to screen and observe the form and distribution of ossification. Therefore, we conducted an observation study focusing on the incidence and structure of minute ossification present in the PLL of 103 autopsy subjects without clinical manifestation of OPLL. Specifically, the various-sized foci in the PLL observed as high-density areas using X-ray analysis were initially screened using serial images of a micro-focus X-ray CT, and the 3D reconstructions were generated to observe the form and continuity with existing bones. The structure of the ossification observed as high-density areas was subjected to a detailed histological examination. In addition, the correlation between the prevalence or form of ossification and a previously known putative predictive factor was assessed, and the potential induction of clinical OPLL through these minute ossifications was reviewed.

Although previous studies using simple cervical X-ray or CT in subjects without clinical symptoms of OPLL have reported that the frequency of ossification detected ranged from 1.9 to 4.3 % [12, 13], the present study revealed a high prevalence of ossifications observed as high-density areas (46.6 %) in the PLL following scanning using micro-focus X-ray CT. This remarkable difference in sensitivity likely reflects differences in the resolution of procedures, namely, in vivo simple X-ray or CT vs. *extra vivo* micro-focus X-ray CT. The aim of the present study was to determine whether the presence of the minute foci in the ossification is a common aspect of the PLL. However, notably a significant correlation was observed between the proportion of the minute high-density area (corresponding to calcification and/or ossification) and both age and glucose intolerance, which have generally been accepted as important predictive factors for clinically overt OPLL [14], suggesting that the development of minute ossifications might be affected through a pathophysiology similar to that of ossifications that induce clinically overt OPLL.

The finding that only the isolated posterior-annular fibrosus type was not significantly correlated with age might suggest that mechanical stress is another important predictive factor for inducing ossification in the PLL, as this area primarily affected with stresses that greatly influence the skeletal form and lifestyle of an individual. In fact, calcification and/or ossification in this type were observed in the thickened ligament of a dorsal portion of the annular fibrosus, which did not have continuity to enthesis. In addition, these findings also support the hypothesis that the nucleus pulposus is herniated into the site of the ruptured annular fibrosus in most cases.

A previous study reported that a potent regional factor causes OPLL, leading to the initial degeneration and subsequent herniation of the nucleus pulposus in ttw mice [15]. Therefore, the rupture of the annular fibrosus based on the degeneration of the disc might play an important role in inducing ossification in this type. However, most cases of the continuous posterior-annular fibrosus type were observed as a projection of enthesis because both the marrow and cortex of the projection of the bone had continuity to pre-existing enthesis, although calcification surrounding the bone as the ossification front was typically present. In the ossification front, degenerative changes in the elastic fibers and cartilaginous cartilage formation are typically observed, together with the appearance of metaplastic hypertrophic cartilage cells and neovascularization [16]. Therefore, we propose that this type of ossification results from the elongation of enthesis rather than the formation of metaplastic bone.

The micro-CT analysis in the present study revealed a third ossification form, the posterior-vertebral type, histologically characterized by a linear-shaped, stereoscopically thin plate-shaped calcification in the PLL developed just behind the vertebral body. The ossification was essentially consistent with calcification, half of which contained islets of mature bone in various degrees. These findings suggested that the bone exhibited in this ossification type might be generated through a dystrophic calcification-bony metaplasia sequence. Therefore, a difference in the mechanism of ossification between both the continuous posterior-annular fibrosus type and the posterior-vertebral type might exist. The former could be regarded as an extension of the existing bone by endochondral ossification. However, because a previous study reported that the PLL behind the vertebral body is less affected through tension stress-related spinal flexion [17], the ossification that developed behind the vertebral body was much less influenced through mechanical stress that might be regarded as a static natural course, such as aging-related changes. Although the elongation of the existing bone has been widely regarded as one of the important courses of OPLL, the results of the present study highlighted the minute ossification that developed in PLL of non-clinically overt OPLL subjects, suggesting another manner of bone formation in PLL, namely, one that has a close relationship to rupturing of the annular fibrosus through mechanical stress and another regarded as dystrophic calcification-based bony metaplasia.

## Conclusion

We observed the PLL in 103 cadavers using micro-focus X-ray CT images and histological sections.

1. Minute foci of calcification and/or ossification greater than 0.1 mm^2^ were observed in the PLL of 48 cases (46.6 %), half of which revealed mature bone structures inside this area (23.3 %).
2. The proportion of minute foci of calcification observed in the present study was correlated with age and glucose tolerance, suggesting that these changes are associated with OPLL in the early stage.
3. Three different mechanisms of ossification were observed: The two ossification structures developed behind the disc might reflect the elongation of enthesis or rupture of annular fibrosus, while the remaining structure developed behind vertebral body might reflect a sequence of dystrophic calcification-based bony metaplasia.

## Limitation

This study was a retrospective study using autopsy cases; thus, some of the values during the extraction of information were missing, reflecting the autopsy protocol and incomplete medical records. Additionally, each numerical value might include a change relative to the condition of the original disease because these values were obtained at death or during the last admission.

In all cases, the spine of the thoracolumbar transition was preserved, and the investigations were therefore performed using the lumbar spine. However, the cervical spine is most frequently affected with OPLL. Furthermore, because we performed tests using micro-focus X-ray CT, the size of the specimens was limited, and only one intervertebral level could be investigated.

## Abbreviations

PLL: posterior longitudinal ligament
OPLL: ossification of the posterior longitudinal ligament
CT: computed tomography

## References

[1] Key C (1838). On paraplegia depending on disease of the ligaments of the spine. Guys Hosp Rep.

[2] Ohtsuka K, Terayama K, Yanagihara M, Wada K, Kasuga K, Machida T (1987). A radiological population study on the ossification of the posterior longitudinal ligament in the spine. Arch Orthop Trauma Surg.

[3] Kobashi G, Washio M, Okamoto K, Sasaki S, Yokoyama T, Miyake Y (2004). High body mass index after age 20 and diabetes mellitus are independent risk factors for ossification of the posterior longitudinal ligament of the spine in Japanese subjects: a case-control study in multiple hospitals. Spine (Phila Pa 1976).

[4] Akune T, Ogata N, Seichi A, Ohnishi I, Nakamura K, Kawaguchi H (2001). Insulin secretory response is positively associated with the extent of ossification of the posterior longitudinal ligament of the spine. J Bone Joint Surg Am.

[5] Shingyouchi Y, Nagahama A, Niida M (1996). Ligamentous ossification of the cervical spine in the late middle-aged Japanese men. Its relation to body mass index and glucose metabolism. Spine (Phila Pa 1976).

[6] Kawamura T, Kinoshita M, Katsushima S, Tokiguchi S (1986). [Myotonic dystrophy with transverse myelopathy caused by ossification of the posterior longitudinal ligament--report of two cases]. Rinsho Shinkeigaku.

[7] Bussiere JL, Ristori JM, Miravet L, Piat C, Soubrier M, Bardin T (1993). Vitamin-resistant hypophosphatemic rickets and spinal cord compression. Apropos of 2 cases. Rev Rhum Ed Fr.

[8] Velan GJ, Currier BL, Clarke BL, Yaszemski MJ (2001). Ossification of the posterior longitudinal ligament in vitamin D-resistant rickets: case report and review of the literature. Spine (Phila Pa 1976).

[9] Okazaki T, Takuwa Y, Yamamoto M, Matsumoto T, Igarashi T, Kurokawa T (1984). Ossification of the paravertebral ligaments: a frequent complication of hypoparathyroidism. Metabolism.

[10] Tsuzuki N, Imai T, Hotta Y (1981). [Histopathological findings of the ossification of the posterior longitudinal ligament of the cervical spine and their significance (author’s transl)]. Nihon Seikeigeka Gakkai Zasshi.

[11] Chen J, Song D, Wang X, Shen X, Li Y, Yuan W (2011). Is ossification of posterior longitudinal ligament an enthesopathy?. Int Orthop.

[12] Tsuyama N (1984). Ossification of the posterior longitudinal ligament of the spine. Clin Orthop Relat Res.

[13] Fujimori T, Le H, Hu SS, Chin C, Pekmezci M, Schairer W (2015). Ossification of the posterior longitudinal ligament of the cervical spine in 3161 patients: a CT-based study. Spine (Phila Pa 1976).

[14] Matsunaga S, Sakou T (2012). Ossification of the posterior longitudinal ligament of the cervical spine: etiology and natural history. Spine (Phila Pa 1976).

[15] Hirakawa H, Kusumi T, Nitobe T, Ueyama K, Tanaka M, Kudo H (2004). An immunohistochemical evaluation of extracellular matrix components in the spinal posterior longitudinal ligament and intervertebral disc of the tiptoe walking mouse. J Orthop Sci.

[16] Sato R, Uchida K, Kobayashi S, Yayama T, Kokubo Y, Nakajima H (2007). Ossification of the posterior longitudinal ligament of the cervical spine: histopathological findings around the calcification and ossification front. J Neurosurg Spine.

[17] Okamoto Y, Yasuma T (1967). Ossification of the posterior longitudinal ligament of cervical spine with or without myelopathy. Nihon Seikeigeka Gakkai Zasshi.

